# Bibliography

**Published:** 1901-01

**Authors:** 


### Bibliography.

THE AMERICAN TEXT-BOOK OF OPERATIVE DENTISTRY. In Contributions by Eminent Authorities. Edited By Edward C. Kirk, D.D.S., Professor of Clinical Dentistry in the University of Pennsylvania, etc. Second Edition, revised and enlarged. Illustrated with Eight Hundred and Ninety-seven Engravings. Lea Brothers & Co., Philadelphia and New York, 1900.

This, in many respects the most valuable work on operative dentistry published in the English language, is presented to the dental profession in its second edition. The fact that this has been required in such a comparatively brief period since the first edition was published, indicates more than words that it was needed in the educational work of dentistry.

There are many objections, however, to this form of cyclopædic presentation as a text-book, and this was noted in the original review of this book by the present writer, but the editor does not regard it as a serious difficulty, for he says in the preface, "The editor is fully aware of the importance which attaches to the work of harmonizing the treatment of the individual subjects and of so co-ordinating them that conflicting views shall not confront the student and thus interfere with his advancement into an untrodden field. . . . Specialization in operative dentistry has not reached the point where it may be deemed best to limit the field of text-book treatment to its technical procedures and relegate the allied subjects to separate volumes."

While this is in part true, it becomes necessary, as each branch develops, to confine it as much as possible to the procedures legitimately belonging to it, hence it would seem that dental embryology is not, strictly speaking, part of operative dentistry, any more than would be comparative anatomy. These subjects, of great value intrinsically, should be considered apart from technical procedures. The same may be said of histology, but there is some reason for the appearance of this valuable paper, inasmuch as operative procedures hold an intimate connection with all the tissues, and their minute structure is of constant importance and must be considered in every operation. The reviewer is of the opinion that the time has come when books must cease to teach everything. Dentistry is becoming more and more specialized, and the question will require an answer in the near future, How may the different subjects be classified to prepare satisfactory text-books? In the present volume large space is given to orthodontia. This is ably handled by a recognized specialist, but it is thought it would have been better had the matter been reduced to a systematic consideration of principles and appliances, for the reason that no branch of dentistry has been more thoroughly or more ably treated in separate volumes than this of regulating teeth. It also occupies the border-line between prosthetic and operative dentistry, and may legitimately be classed with both. While the limiting, or removal, of these would lessen bulk, an important matter, it would have given room for the admission of a chapter on artificial crowns. This is mainly an operative procedure, and in some respects entirely so. The preparation of the canals so much belongs to the operative side, and is so vitally important, that it seems wrong to relegate it wholly to the mechanical branch of dentistry. The division might properly be made between the insertion of single crowns and bridge-work, the latter strictly belonging to prosthetic dentistry.

With the exception of the chapter on "Dental Histology," profusely illustrated, and that on "Antisepsis in Dentistry," there are no new chapters, but all have undergone revision by the various authors, so that on special practical subjects they are all brought up to the present period.

The readers of this volume must regret the great loss sustained in the death of one of the original and principal contributors, Dr. Burchard, for through this sacrifice the work he did upon the first edition has failed to receive his careful revision, and still evidences the need of a more extended practical experience than was originally given it. Under the present circumstances criticism would be entirely out of place, but the reviewer cannot avoid calling the editor's attention, on page 471, to the use of a ten per cent. formalin solution. It is well known that an excess of one per cent. is too irritating where such an effect is not to be desired.

There is a very common error made very frequently by writers, and noticeable here, in making use of the expression, "As pointed out by Dr. ——." Now, the subject-matter may be as old as dentistry, but it leads to the impression that the individual mentioned was the discoverer or originator of the process. This makes wrong history, and has been the source of much confusion in questions of fact, as well as much misplaced honor.

The author of the chapter on "Pyorrhœa Alveolaris" still adheres with partisan pertinacity to his conception of the etiology of pyorrhœa, and hence devotes large space to the consideration of gouty conditions and calcic accretions, due to this diathesis, upon the teeth. He fails to give other possible causes for this disease, and almost entirely ignores the possibilities of local irritations. In view of the extended investigations of many, especially those instituted by Dr. Talbot, some notice should have been given these, made both at home and abroad. The influence of bacteria is given but a single paragraph. It does not appear that he regards these as having any near relation to the origin of pyorrhœa, a very unfortunate error, it seems to the reviewer, for the student reading this book will naturally be impressed with the fact that gout, in its protean forms, is the sole cause of this disease. The writer, with an extended experience, has failed to find that gout has any direct influence in producing pyorrhœa; in fact, it can be cured without any systemic treatment in those afflicted.

Objection is also made to the author's limitation of age. He says, "It is rarely seen before the age of thirty, and still less frequently does it make its appearance after the age of sixty." This is certainly an error, for the writer has frequently met with it at twenty, and it is quite common after sixty; in fact, the conditions all favor it after that age, but it is due then to causes which space will not permit the reviewer to enlarge upon.

The objections made here and there in this review are not brought out prominently in a hypercritical spirit, for the book is regarded, as a whole, as a type of that advanced dentistry that belongs to and is a part of that work at the close of the nineteenth century. It thoroughly exhibits upon every page the advance made in operative dentistry since the close of the first half of the century.

The editor can be congratulated upon the care taken to avoid conflict of opinion between the various contributors, a serious task where so few agree upon practical methods.

Dentistry owes a debt of gratitude to Lea Brothers & Co., that they have been willing to risk large expenditure in the publishing of the dental series, and it is a satisfaction to feel that this outlay has resulted in no loss to them and has proved of incalculable benefit to the dental profession.

The illustrations are profuse, and fully maintain the character of the first edition.

Treatment of Malocclusion of the Teeth and Fractures of the Maxillæ. Angle's System. Sixth Edition, greatly enlarged and entirely rewritten. With Two Hundred and Ninety-nine Illustrations. By Edward H. Angle, M.D., D.D.S., former Professor of Histology, Orthodontia, and Comparative Anatomy of the Teeth in the Dental Department of the University of Minnesota, etc. The S. S. White Dental Manufacturing Company, Philadelphia, 1900.

This volume, the sixth edition of this valuable work of the author, has been so much improved that it almost assumes the character of an original production. The author says of it, "The subject is treated far more comprehensively in this edition than in those which have preceded it, they having been limited to the mechanical phases of the subject. . . . It has long been the effort of the author to perfect a system which should be complete within itself. . . . The degree of his success must be determined somewhat by time and the intelligent unbiassed judgment of others."

It has been the hope of many minds interested in orthodontia that at some future time a system would be devised that would largely eliminate the necessity for inventing an apparatus for every given case. To this end Dr. Angle has devoted years of work, and that he has, in a large degree, succeeded must be acknowledged by every careful reader of this book. When the writer first began to teach methods of regulating teeth he was met at the very threshold of his inquiry with the necessity of a system, and it seemed to him that, while the presentations of irregularity appeared in many forms, they, nevertheless, seemed to follow one general law and became amenable to one general method of treatment. That Dr. Angle recognized this, and sought a mechanical way out of the difficulty, deserves, and should receive, the highest commendation. Whether his methods are the best possible is quite another question, but to the writer they seem to meet generally all demands. Their greatest merit must be found in the entire elimination of the old and clumsy apparatus connected with plates so much in vogue at an earlier day of practice.

The author contends "that occlusion is the very basis of the science," and, therefore, he makes, in the pages that are to follow, "occlusion the central thought, and on it bases the classification of malocclusion, . . . and will define orthodontia . . . as being that science which has for its object the correction of malocclusion of the teeth."

The importance of correct facial lines is quite fully elaborated in the chapter on "Facial Art," leading up to "etiology of malocclusion," and in Chapter IV. to "Classification and Diagnosis of Malocclusion." Upon this classification, which seems to the writer to cover very nearly all presentable cases, is based the system of treatment.

Chapter V., on "Alveolus and Peridental Membrane," is a clear statement, beautifully illustrated. The important considerations connected with this membrane, in the movement of teeth, is too generally neglected by practitioners, and it is, therefore, a satisfaction to find that the author dwells upon the changes produced by pressure. He might have gone farther and explained the resulting effect of a long-continued and uninterrupted force. As the reviewer reads this book it seems to lack in this essential point, that while it shows how to do the work, it fails to warn the operator sufficiently that a non-intelligent use of appliances may lead to serious pathological conditions.

The author makes use, mainly, of German silver in the preparation of appliances, for the reason, "It is very susceptible to skilful working, and may be developed to possess great strength and rigidity as demanded by the jack and traction screws."

The "Author's Appliances" and the following chapter on "Soldering" are exceedingly valuable as a guide to the student and practitioner. That portion relating to clamp bands is especially good, as it furnishes a ready method of meeting a practical difficulty.

The operation of "Immediate Movement" does not appeal to the author's judgment, for he remarks, "It is a practice as inexcusable and impracticable as it is barbarous." In this opinion the majority of operators must coincide. He, however, advocates the "Resection of Peridental Fibres,"—that is, severing the fibres of the peridental membrane prior to attempting movements of teeth.

The reviewer must regard this as equally barbarous with that of torsion by force, and to his mind wholly unnecessary.

The author favors early regulation for the reason that "the position of teeth may be more readily changed in early childhood (from seven to twelve years of age) than at any other period."

The author has very little regard for the plan of extraction for the purpose of regulating. He contends "that it is difficult to lay down any precise rule regarding extraction, but it is a matter which involves the broadest consideration and closest study of each case, often taxing the judgment as much as does any problem in orthodontia."

It is impossible to follow the author through all the practical pages he presents to the reader. The dental profession is quite familiar with Dr. Angle's appliances, and their extended use is the best evidence that he has succeeded in impressing his methods upon the minds of a very large number of practitioners. The reviewer, however, cannot agree with him in his opinion that it is practically waste time to teach the manufacture of regulating appliances to the average student. To the writer it seems as though this should be the most valuable training in prosthetic dentistry. It is true that not all are capable of making appliances with skill, but all can acquire the knowledge of how these should be made, and this is quite as important as the ability to manufacture.

The most valuable portion of the work of Dr. Angle, as the writer views it, is the fact that he has, to a large degree, perfected a system of regulating teeth; and while we may differ with him in some respects, the main fact still remains that he has accomplished a great work in leading the dental mind into correct methods of diagnosis and practice. This the sixth edition of his work is, therefore, cordially recommended to every practitioner of dentistry for both its practical and its intelligent conception of what is necessary in the movement of teeth.

The book is presented with the usual care and taste of the S. S. White Dental Manufacturing Company.

